# High Genetic Diversity but Absence of Population Structure in Local Chickens of Sri Lanka Inferred by Microsatellite Markers

**DOI:** 10.3389/fgene.2021.723706

**Published:** 2021-09-28

**Authors:** Amali Malshani Samaraweera, Ranga Liyanage, Mohamed Nawaz Ibrahim, Ally Mwai Okeyo, Jianlin Han, Pradeepa Silva

**Affiliations:** ^1^Department of Animal Science, Faculty of Animal Science and Export Agriculture, Uva Wellassa University, Badulla, Sri Lanka; ^2^Postgraduate Institute of Agriculture, University of Peradeniya, Peradeniya, Sri Lanka; ^3^International Livestock Research Institute (ILRI), Nairobi, Kenya; ^4^CAAS-ILRI Joint Laboratory on Livestock and Forage Genetic Resources, Institute of Animal Science, Chinese Academy of Agricultural Sciences (CAAS), Beijing, China; ^5^Department of Animal Science, Faculty of Agriculture, University of Peradeniya, Peradeniya, Sri Lanka

**Keywords:** local chicken, microsatellite marker, genetic diversity, population structure, tropical climate

## Abstract

Local chicken populations belonging to five villages in two geographically separated provinces of Sri Lanka were analyzed using 20 microsatellite markers to determine the genetic diversity of local chickens. Population genetic parameters were estimated separately for five populations based on geographic locations and for eight populations based on phenotypes, such as naked neck, long legged, crested or crown, frizzle feathered, Giriraj, commercial layer, crossbreds, and non-descript chicken. The analysis revealed that there was a high genetic diversity among local chickens with high number of unique alleles, mean number of alleles per locus (MNA), and total number of alleles per locus per population. A total of 185 microsatellite alleles were detected in 192 samples, indicating a high allelic diversity. The MNA ranged from 8.10 (non-descript village chicken) to 3.50 (Giriraj) among phenotypes and from 7.30 (Tabbowa) to 6.50 (Labunoruwa) among village populations. In phenotypic groups, positive inbreeding coefficient (*F*_IS_) values indicated the existence of population substructure with evidence of inbreeding. In commercial layers, a high expected heterozygosity He = 0.640 ± 0.042) and a negative *F*_IS_ were observed. The positive *F*_IS_ and high He estimates observed in village populations were due to the heterogeneity of samples, owing to free mating facilitated by communal feeding patterns. Highly admixed nature of phenotypes was explained as a result of rearing many phenotypes by households (58%) and interactions of chickens among neighboring households (53%). A weak substructure was evident due to the mating system, which disregarded the phenotypes. Based on genetic distances, crown chickens had the highest distance to other phenotypes, while the highest similarity was observed between non-descript village chickens and naked neck birds. The finding confirms the genetic wealth conserved within the populations as a result of the breeding system commonly practiced by chicken owners. Thus, the existing local chicken populations should be considered as a harbor of gene pool, which can be readily utilized in developing locally adapted and improved chicken breeds in the future.

## Introduction

The choice of chickens by rural small-scale poultry farmers in Sri Lanka is often indigenous chickens for both egg and meat production, owing to numerous advantages they bring, such as disease resistance, high fertility, good maternal quality, longevity, ability to utilize poor-quality feeds, and most, importantly, the ability to manage them under a harsh environment condition with low level of management inputs despite their poor productivity compared with commercial layers and broilers (Silva et al., 2014). Moreover, high ash content in eggs of some phenotypes (non-descript village chicken, naked neck, long legged, and crown) and high fat content in egg yolk of local chickens in Sri Lanka were reported in a study by Sanjeewa et al. (2011). In the same study, the internal egg quality was found to be preserved in local chicken eggs during storage compared with commercial chicken eggs (Sanjeewa et al., 2011). In Korean native chicken, considerable amount of health- promoting compounds, such as carnosine, anserine, betaine, and carnitine, were identified compared with commercial broilers (Jayasena et al., 2015). Therefore, there is a special preference for native chicken meat due to its unique taste and texture, especially after cooking (Wattanachant et al., 2004).

However, such valuable indigenous genetic pool with undiscovered genetic potential has been eroding due to various reasons, especially in developing counties. Furthermore, some of the poultry breeds in Sri Lanka have already been lost or are at the risk of extinction (Punyawardena, 2010). Conservation of genetic resources is important from a global perspective as genetic variability underlying the adaptability and potential of animal genetic resources is essential to meet the changes in the preference and demand of consumers and to diminish the challenges posed by climate change and emerging diseases (FAO, 2007). Hence, it is frequently highlighted that the characterization of animal genetic resources is an essential initiative for sustainable utilization of animal genetic resources (AnGR). The Global Strategy for the Management of Farm Animal Genetic Resources coordinated by the Food and Agricultural Organization (FAO) aims to identify and propose sustainable genetic improvement plan for indigenous AnGR. Characterizing AnGR both phenotypically and genotypically is essential to understand and describe it properly, and then to propose a rational action plan for sustainable utilization. Accordingly, the attempts made on phenotypic characterization of indigenous chickens in Sri Lanka identified that the populations consisted of diverse phenotypes (Bett et al., 2014), while Liyanage et al. (2015) described seven distinct phenotypic groups, including naked neck, long legged, crest/crown, Giriraj, commercial crosses, frizzle feathered, and the non-descript group of multiple crosses of other groups.

So far, a large number of studies have been conducted to characterize the chicken populations in Asia and Africa using microsatellite markers due to their highly polymorphic nature and abundance throughout the entire genome (Berthouly et al., 2009; Bodzsar et al., 2009; Cuc et al., 2010; Eltanany et al., 2011; Leroy et al., 2012). With recent advances in DNA sequencing, single-nucleotide polymorphisms (SNPs) have been used extensively to characterize the genetic diversity and animal identification systems for closely related species/breeds/types (Samaraweera et al., 2011). However, since SNPs are biallelic and less informative, around 1.7–5.56 SNPs were needed to achieve the same information content as that obtained with one microsatellite marker (Gärke et al., 2012). Moreover, if short tandem repeats (STRs) have been selected based on a high minor allele frequency in a restricted number of breeds, this may underestimate the diversity in other breeds (Lenstra et al., 2012). Therefore, the highly polymorphic microsatellite markers are still valid for assessing the genetic diversity of AnGR.

In this context, this study was conducted to determine the genetic diversity of local chicken populations in two selected areas and among identified phenotypes in Sri Lanka using microsatellite markers.

## Materials and Methods

### Selection of Sampling Locations and Households

**FIGURE 1:**
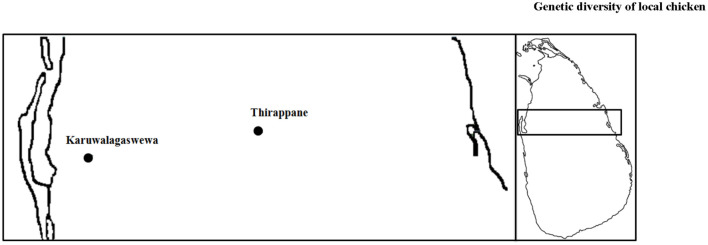
The Sri Lankan map showing the two sampling locations, Thirappane and Karuwalagaswewa.

Two veterinary divisions, Thirappane (80.5039–80.6331 E, 8.1185–8.2202 N) in the North Central Province and Karuwalagaswewa (79.5395–80.5042 E, 8.0047–8.0692 N) in the North Western Province were selected for sample collection based on the highest density of local chickens in Sri Lanka (Figure 1). According to the distribution of farms, three villages of the Thirappane site, namely, Dematagama (DM), Labunoruwa (LA), and Ooththupitiya (OT), and two villages in the Karuwalagaswewa site, namely, Tabbowa (TB) and Thewanuwara (TH), were sampled.

### Sample Collection

Altogether 818 blood samples of chickens older than 6 months of age were collected on the Whatman FTA^®^ filter paper (Whatman Bio-Science, Maidstone, United Kingdom) and stored at room temperature until it was used for the analysis (i.e., 216, 69, 67, 219, and 247 from DM, LA, OT, TB, and TH, respectively). Among 818 samples, 192 samples were purposively selected for genotyping based on locations, households, and phenotypes (Table 1). Ethical permission for the project was obtained from the Institutional Research and Ethics Committee (IREC) and the Institutional Animal Care and Use Committee (IACUC) of the International Livestock Research Institute (ILRI), Nairobi, Kenya.

**TABLE 1:** The number of village chicken ecotypes selected from each household of the five villages in two sites (Thirappane and Karuwalagaswewa) for genotyping analysis.

Site	Village	Village chicken (VC)	Naked neck (NN)	Long leg (LL)	Crown (CC)	Frizzled feathered (FF)	Giriraj (GR)	Commercial layers (CL)	Cross (CR)	Total number of birds from each village
Thirappane (Site 1)	Dematagama (DM)	10	10	6	4	1	0	6	0	37
	Labunoruwa (LA)	19	8	0	2	3	5	2	0	39
	Ooththupitiya (OT)	20	17	3	0	0	0	0	0	40
Karuwalagaswewa (Site 2)	Tabbowa (TB)	12	13	12	0	1	0	0	1	39
	Thewanuwara (TH)	13	7	10	0	1	0	0	6	37
Total		74	55	31	6	6	5	8	7	192

DNA extraction and genotyping using microsatellite markers were carried out at the CAAS-ILRI Joint Laboratory on Livestock and Forage Genetic Resources, Institute of Animal Science, CAAS, Beijing, China.

### DNA Extraction, PCR Amplification, and Genotyping

DNA extraction was carried out from blood collected on the FTA filter paper, and around 15 discs from each FTA filter paper were added to 100 μl of distilled water and then heated at 90°C for 10 min. The resulting solution was used for PCR amplification with 20 microsatellite markers recommended by the International Society of Animal Genetics (ISAG)–FAO Advisory Group on Animal Genetic Diversity (Table 2; FAO, 2011). The PCR amplicons were separated by size using the 3130xl Genetic Analyzer (Applied Biosystems, Carlsbad, CA, United States). Sizing and genotyping of the alleles were carried out using the GeneMapper 3.7 software (Applied Biosystems).

**TABLE 2:** Primer name, primer sequence, allele size, and annealing temperature of 20 microsatellite markers used for the analysis.

	Name	Primer sequence (5′ - > 3′) forward reverse	Label	Allele range (bp)	Annealing temperature (°C)
1	LEI0094	GATCTCACCAGTATGAGCTGC TCTCACACTGTAACACAGTGC	FAM	240–300	58.5
2	MCW0069	GCACTCGAGAAAACTTCCTGCG ATTGCTTCAGCAAGCATGGGAGGA	HEX	150–180	
3	ADL0268	CTCCACCCCTCTCAGAACTA CAACTTCCCATCTACCTACT	FAM	100–120	53
4	MCW0034	TGCACGCACTTACATACTTAGAGA TGTCCTTCCAATTACATTCATGGG	HEX	210–250	
5	LEI0166	CTCCTGCCCTTAGCTACGCA TATCCCCTGGCTGGGAGTTT	FAM	340–380	60
6	MCW0248	GTTGTTCAAAAGAAGATGCATG TTGCATTAACTGGGCACTTTC	FAM	210–230	
7	MCW0216	GGGTTTTACAGGATGGGACG AGTTTCACTCCCAGGGCTCG	FAM	135–150	
8	LEI0234	ATGCATCAGATTGGTATTCAA CGTGGCTGTGAACAAATATG	HEX	210–370	54.3
9	ADL0278	CCAGCAGTCTACCTTCCTAT TGTCATCCAAGAACAGTGTG	HEX	110–130	
10	MCW0222	GCAGTTACATTGAAATGATTCC TTCTCAAAACACCTAGAAGAC	FAM	200–230	55
11	MCW0016	ATGGCGCAGAAGGCAAAGCGATAT TGGCTTCTGAAGCAGTTGCTATGG	HEX	135–160	
12	MCW0295	ATCACTACAGAACACCCTCTC TATGTATGCACGCAGATATCC	FAM	85–110	54.3
13	MCW0037	ACCGGTGCCATCAATTACCTATTA GAAAGCTCACATGACACTGCGAAA	FAM	150–160	
14	MCW0206	CTTGACAGTGATGCATTAAATG ACATCTAGAATTGACTGTTCAC	FAM	220–250	58.6
15	MCW0111	GCTCCATGTGAAGTGGTTTA ATGTCCACTTGTCAATGATG	FAM	95–115	
16	MCW0067	GCACTACTGTGTGCTGCAGTTT GAGATGTAGTTGCCACATTCCGAC	HEX	150∼200	56
17	MCW0183	ATCCCAGTGTCGAGTATCCGA TGAGATTTACTGGAGCCTGCC	FAM	290–330	
18	MCW0014	TATTGGCTCTAGGAACTGTC GAAATGAAGGTAAGACTAGC	FAM	160–200	54
19	MCW0330	TGGACCTCATCAGTCTGACAG AATGTTCTCATAGAGTTCCTGC	HEX	250–300	
20	MCW0081	GTTGCTGAGAGCCTGGTGCAG CCTGTATGTGGAATTACTTCTC	FAM	100∼120	54.6

### Data Analysis

**FIGURE 2:**
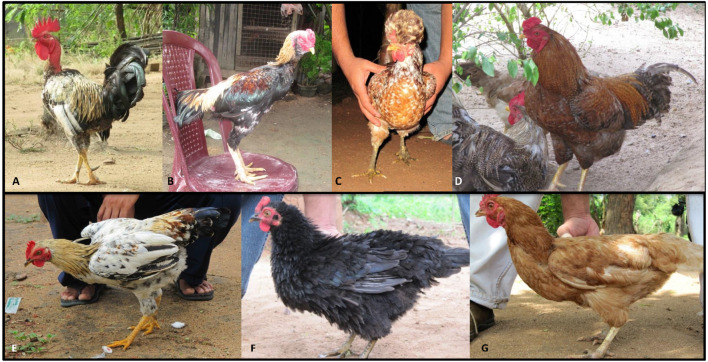
Chicken phenotypes in Sri Lanka. **(A)** Naked neck; **(B)** long legged; **(C)** crested or crown; **(D)** Giriraj; **(E)** non-descript chicken; **(F)** frizzle feathered; **(G)** commercial layer.

The total number of samples used in the analysis was divided into five populations according to the geographical locations (villages) of sampling as DM, LA, OT, TB and TH (Supplementary Table 1), and to eight phenotypic groups; Naked Neck (NN), Long Legged (LL), Crested or Crown (CC), Frizzle Feathered (FF) Giriraj (GR), Commercial Layer (CL), Crossbreds (CR) and non-descript village chicken (VC) (Figure 2, Supplementary Table 2).

Deviations from Hardy–Weinberg equilibrium (HWE) for all locus-population combinations and linkage disequilibria (LD) between all pairs of loci were determined using the program Genepop version 4.1.3 (Raymond, 1995). HWE was assessed based on the Markov chain algorithm to estimate the unbiased exact *p*-value of the test (Guo and Thompson, 1992) for each locus in each population. Fisher’s exact test was used to test for LD with unbiased *p*-values derived from the Markov chain randomization method.

Allelic frequency, mean number of alleles per locus (MNA), observed heterozygosity (Ho), and expected heterozygosity (He) were calculated to quantify the genetic variation within populations using Microsatellite Toolkit version 3.1 (Park, 2001) and FSTAT version 2.9.3.2. MNA is the average number of alleles observed in a population over all loci genotyped. F-statistics for each locus (Weir and Cockerham, 1984) was calculated and tested using the FSTAT.

Population structure and the level of admixture of individuals were analyzed using the Structure version 2.3.4 (Pritchard et al., 2000). Population structure was analyzed assuming the most probable number of the clusters (*K*) values at 1–6 with 30 simulations for each *K*, assuming an admixture model with correlated allele frequencies using a burn-in of 100,000 followed by 100,000 Markov Chain Monte Carlo iterations to check the level of admixture and substructuring of the populations. The most probable number of the clusters (*K*) was selected based on the highest averaged log probability of data [Ln Pr(*X/K*)] with the lowest variation {Var[Ln Pr(*X/K*)]} among the 30 runs. *Ad hoc* quantity (Δ*K*) based on the rate of change in the log probability of data between successive *K* values was calculated to verify the best *K* using the web-based STRUCTURE HARVESTER (Earl, 2012). Estimated cluster membership coefficient matrices of multiple runs for each *K* were permuted using the CLUMPP (Jakobsson and Rosenberg, 2007) to obtain close match in all simulations with the Greedy algorithm at 1,000 repeats of random inputs of the data and the pairwise matrix similarity statistic to be G′. Then the aligned membership coefficients were displayed using Microsoft Office Excel 2007. Principal component analysis (PCA) was performed using the SPSS version 13.0^1^ with arcsine-transformed allele frequencies. Factorial correspondence analysis (FCA) was performed using GENETIX 4.05 (Belkhir, 2004).

An unweighted pair-group method using arithmetic average (UPGMA) dendrogram between populations was constructed from Nei’s standard genetic distances (*D*_S_; Nei, 1987) using DISPAN (Ota, 1993) with 1,000 bootstrap values. Analysis of molecular variance (AMOVA), pairwise *F*_ST_ (Reynolds’ genetics distance), and correlation between distance matrices (Mantel test with 10,000 permutations) were computed using Arlequin version 3.5.2.2 (Excoffier et al., 2005). The Splits Tree4 version 4.14.2 was used to draw a NeighborNet tree based on pairwise *F*_ST_ values (Huson and Bryant, 2006).

## Results

The present study assessed the genetic diversity and genetic structure within Sri Lankan local chicken populations, which have been defined using two different boundaries of geographical boundaries (five villages) and phenotype-based boundaries (eight phenotypes). The results are also presented accordingly.

### Hardy–Weinberg Equilibrium and Linkage Disequilibria in Different Local Chicken Populations

It was observed that among 20 loci across five geographic populations used in the analysis, several loci in individual populations (4 from DM, 8 from LA, 10 from OT and 6 each from TB and TH) were deviated from HWE (*p* < 0.05) while no pair of loci rejected LD (*p* > 0.05).

### Genetic Diversity

#### Village Populations

From 818 samples, more than 15% of birds in each of the five villages were selected for the genotyping analysis. Accordingly, 192 samples across five geographic populations were genotyped, and a total of 185 microsatellite alleles were detected at 20 loci, indicating a considerably rich allelic diversity. The number of alleles across loci within populations ranged from 130 alleles in LA to 146 alleles in TB. The number of alleles per locus per population ranged from 3 (MCW0248 in DM; MCW0014 in LA) to 16 (LEI0234 in DM). Moreover, 27 private alleles, which were unique to one population, were found among five populations, e.g., 6 in DM, 4 in LA, 6 in OT, 9 in TB, and 2 in TH. Furthermore, both the mean frequency of individual private alleles across the five populations (1.97%) and the population-specific frequency of the private alleles (1.3%) were very low, including a high number of migrants per population (8.33), similar to what was reported by Barton and Slatkin (1986).

Estimates of *F*_IS_ indicates important properties of the mating system within populations (Holsinger and Weir, 2009). Accordingly, a deficit of heterozygotes (positive *F*_IS_ values; Tables 3, 4) was observed for all populations, showing certain levels of inbreeding.

**TABLE 3:** Mean number of alleles per locus (MNA), expected heterozygosity (He), observed heterozygosity (Ho), and average inbreeding coefficient (*F*_IS_) estimated from the 20 microsatellite loci for each village population.

Village	n	MNA	He	Ho	*F*_*IS*_
DM	37	7.10 ± 3.35	0.715 ± 0.023	0.689 ± 0.017	0.036
LA	39	6.50 ± 2.48	0.678 ± 0.027	0.594 ± 0.018	0.126
OT	40	7.05 ± 2.65	0.709 ± 0.024	0.628 ± 0.017	0.116
TB	39	7.30 ± 2.74	0.715 ± 0.022	0.653 ± 0.017	0.088
TH	37	7.10 ± 2.53	0.706 ± 0.020	0.635 ± 0.018	0.102

**TABLE 4:** Mean number of alleles per locus (MNA), expected heterozygosity (He), observed heterozygosity (Ho), and average inbreeding coefficient (*F*_IS_) estimated from the 20 microsatellite loci for each phenotype.

Phenotype	n	MNA	He	Ho	*F*_*IS*_
VC	74	8.10 ± 2.94	0.714 ± 0.021	0.626 ± 0.013	0.123
NN	55	7.80 ± 3.27	0.716 ± 0.023	0.646 ± 0.014	0.099
LL	31	6.75 ± 2.55	0.722 ± 0.025	0.686 ± 0.019	0.051
GR	5	3.50 ± 1.36	0.611 ± 0.044	0.580 ± 0.049	0.051
FF	6	3.90 ± 1.29	0.656 ± 0.041	0.650 ± 0.043	0.009
CR	7	4.40 ± 1.57	0.662 ± 0.040	0.557 ± 0.042	0.158
CL	8	4.30 ± 1.26	0.640 ± 0.042	0.650 ± 0.038	–0.016
CC	6	3.70 ± 1.26	0.652 ± 0.035	0.625 ± 0.044	0.041

The MNA per locus for populations ranged from 6.5 in LA to 7.3 in TB. Therefore, a high MNA per locus was observed, indicating a high level of polymorphisms. MNA is also a sensitive measure of genetic variability in comparison with heterozygosity measures. Ho ranged from 0.594 in LA to 0.689 in DM, while He ranged from 0.678 in LA to 0.715 in DM.

#### Phenotype-Based Populations

From 818 samples, more than 12% of birds from each of the eight phenotypes were selected for the genotyping analysis. The number of alleles across loci in the eight phenotypes ranged from 162 in CR to 70 in FF. A total of 29 private alleles were detected in VC (12), NN (13), LL (3), and CL (1). The MNA per locus per phenotype ranged from 8.1 in VC to 3.5 in GR (Table 4).

### Genetic Differentiation

The genetic relationships between populations were determined using Reynolds’ genetic distances assuming that the genetic differentiation occurs solely due to genetic drift (Reynolds et al., 1983).

#### Village Populations

**FIGURE 3:**
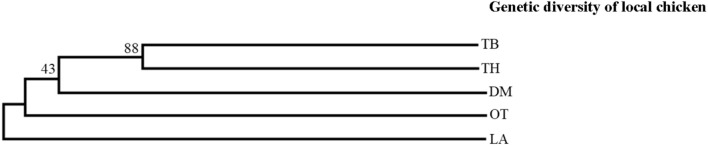
The unweighted pair-group method using arithmetic averages (UPGMA) dendrogram summarizing the standard genetic distances (*Ds*) between village-based populations. The numbers at the nodes are the percentage bootstrap values after 1,000 replications over loci.

The two villages of TB and TH at Karuwalagaswewa clustered into one group separating other three villages (DM, LA, and OT) at Thirappane. The largest (0.0240) and the smallest (0.0090) pairwise genetic distances were observed between TH and OT and between TH and TB, respectively (Table 5). Furthermore, Figure 3 shows the unrooted UPGMA dendrogram summarizing the Nei’s standard genetic distances between villages, confirming the differentiation in local chickens between the two veterinary divisions with a high bootstrap value at 88%. The result of AMOVA for villages is given in Table 6, in which most of the genetic variability based on the 20 microsatellite markers (89%) was found to be present among alleles within individuals, followed by the one observed among individuals within the villages (9%).

**TABLE 5:** Pairwise *F*_*ST*_ estimates between the five chicken populations based on the 20 microsatellite loci.

Populations	DM	OT	LA	TB	TH
DM					
OT	0.0161				
LA	0.0184	0.0219			
TB	0.0128	0.0123	0.0235		
TH	0.0160	0.0240	0.0216	0.0090	

**TABLE 6:** Analysis of molecular variance (AMOVA) based on 20 microsatellite loci of five populations (villages) assigned into two groups (two sites).

Sources of variation	df	Percentage of variation
Between sites	1	0.34
Between villages, within site	3	1.43
Between individuals, within village	187	9.21
Within individuals	192	89.02

#### Phenotype-Based Populations

**FIGURE 4:**
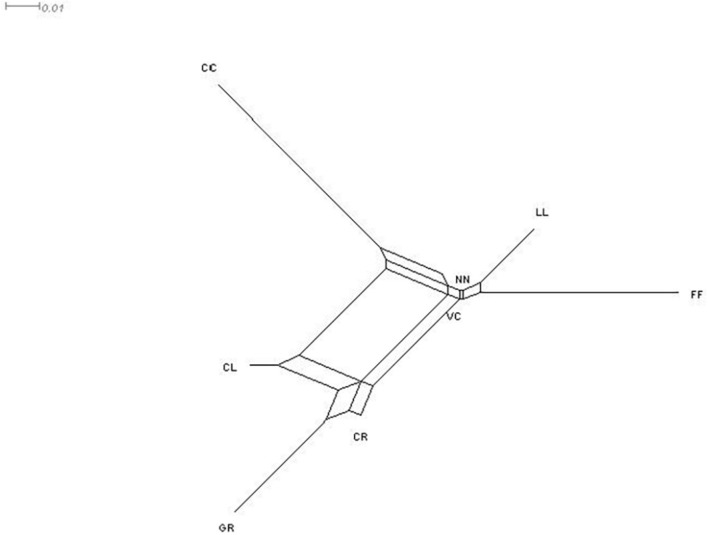
NeighborNet tree summarizing the genetic distances between chicken phenotypes. NN, naked neck; LL, long legged; CC, crested or crown; FF, frizzle feathered; GR, Giriraj, CL, commercial layer; CR, crossbreds; VC, non-descript chicken.

The highest genetic distance (Reynolds’ genetic distances in Table 7) was observed between GR and CC (0.0914), but the lowest was between the NN and VC (0.0023). The relationship between the eight phenotypes is presented in Figure 4 as a NeighborNet tree derived from pairwise *F*_ST_ estimates. Two main clusters were identified, where VC, NN, LL, and FF, were clustering together, while CL, GR, and CR formed a separate cluster, leaving CC as a unique phenotype. Similarly, the highest genetic distance in CC to other phenotypes was found in NeighborNet tree (Figure 4). The result of AMOVA for phenotypes is given in Table 8, in which the highest genetic variability (89%) was found among alleles within individuals, followed by that present among individuals within the phenotypes.

**TABLE 7:** Pairwise *F*_ST_ estimates between the eight chicken phenotypes based on the 20 microsatellite loci.

	VC	NN	LL	CC	FF	GR	CL	CR
VC								
NN	0.0023							
LL	0.0115	0.0121						
CC	0.0280	0.0263	0.0488					
FF	0.0175	0.0301	0.0323	0.0842				
GR	0.0446	0.0364	0.0475	0.0914	0.0843			
CL	0.0326	0.0330	0.0496	0.0602	0.0570	0.0401		
CR	0.0173	0.0200	0.0280	0.0663	0.0471	0.0171	0.0244	

**TABLE 8:** Analysis of molecular variance (AMOVA) based on the 20 microsatellite loci of eight populations (phenotypes).

Sources of variation	df	Percentage of variation
Between phenotypes	7	1.5
Between individuals, within phenotype	184	9.4
Within individuals	192	89.1

**FIGURE 5:**
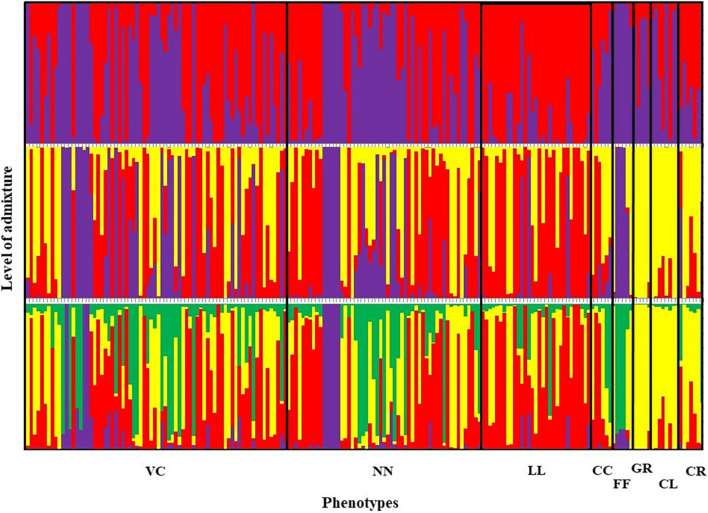
Summary bar plot of estimates of Q (the estimated membership coefficient for each individual in each cluster). Each individual is represented by a single vertical line broken into *K* colored segments with lengths proportional to *K* inferred clusters (best *K* = 2). Chicken phenotypes: NN, naked neck; LL, long legged; CC, crested or crown; FF, frizzle feathered; GR, Giriraj, CL, commercial layer; CR, crossbreds; VC, non-descript chicken.

Furthermore, a high genetic similarity between NN and VC was further confirmed by the population structure analysis (Figure 5). The structure analysis indicated that VC, NN, LL, CC, and FF shared a higher proportion of genotypes in common compared with GR, CL, and CR.

### Population Structure

**FIGURE 6:**
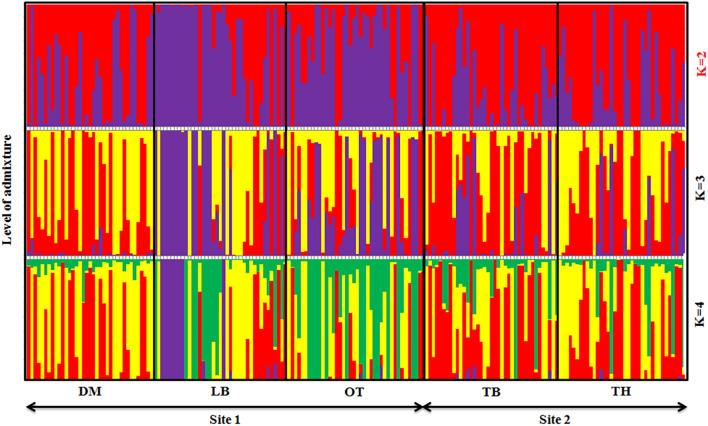
Summary bar plot of estimates of Q (the estimated membership coefficient for each individual in each cluster). Each individual is represented by a single vertical line broken into *K* colored segments with lengths proportional to *K* inferred clusters (best *K* = 2).

**FIGURE 7:**
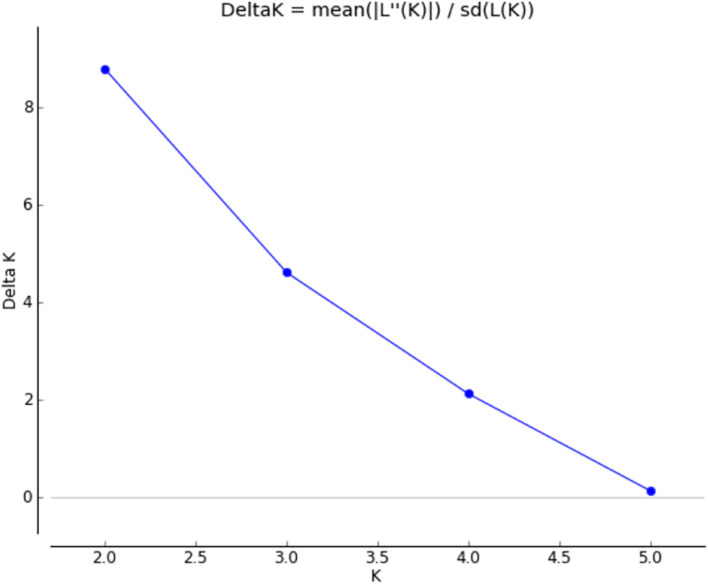
Graphical illustration of delta *K*.

Graphic displays of the estimated membership coefficients of each individual to each population based on the phenotypes and based on villages at 2 ≤ *K* ≥ 4 are given in Figures 5, 6, respectively. The STRUCTURE HARVESTER software was used to graphically illustrate the mean estimates of log probability of data and Δ*K* to select the best *K* value (Figure 7). Accordingly, the rate of change in the log probability of data between successive *K* values (Δ*K*) against each *K* indicated that a higher Δ*K* value at *K* = 2. Therefore, *K* = 2 was selected as the most probable number of the clusters to reveal the population structure in both situations. Structure analysis indicated the absence of population genetic structure among local chicken populations in Sri Lanka.

**FIGURE 8:**
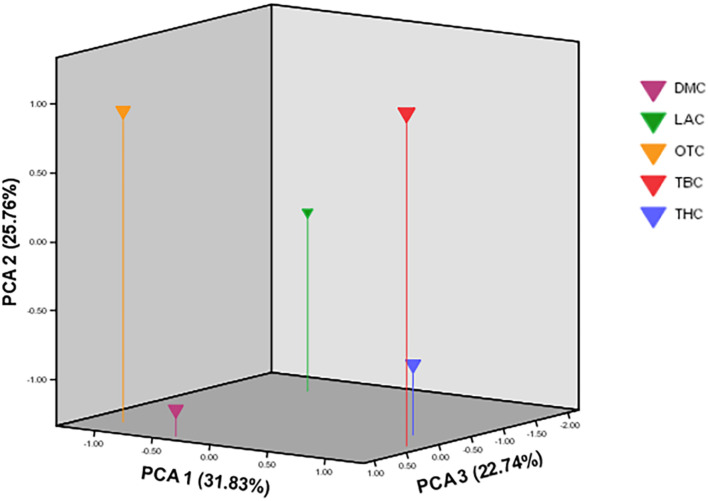
Scatter plot showing the first three principal components over five village-based populations analyzed as revealed by principal component analysis (PCA) implemented in MVSP (Populations: DM, Dematagama; LA, Labunoruwa; OT, Ooththupitiya; TB, Tabbowa; TH, Thewanuwara).

**FIGURE 9:**
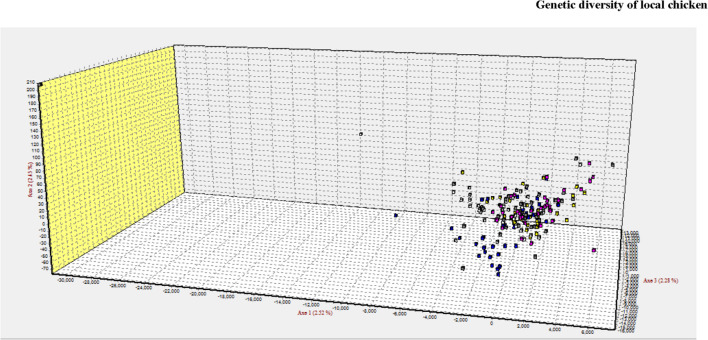
3D clustering patterns of all individuals analyzed using 20 microsatellite markers as revealed by factorial correspondence analysis (FCA) implemented in GENETIX.

Compared with the phylogenetic reconstruction methods, PCA provided a better understanding of the genetic relationship among the local chicken populations, precisely the level of admixture (Figure 8). Similar to the structure result, the FCA analysis also indicated the absence of population structure (Figure 9).

## Discussion

The present study investigated the genetic diversity and population genetic structure of two categories of indigenous chicken populations defined by geographical boundaries and phenotypic boundaries in two geographically distant regions of Sri Lanka.

The result showed that some loci were deviated from HWE, probably due to the deficiencies of heterozygotes. Several factors, like the within-population fragmentation created by clusters of households, which were involved in our stratified sampling (Samaraweera et al., 2014), could bring the Wahlund effect, leading to an overall deficiency of heterozygotes. The inbreeding observed in all five village chicken populations (Table 3) could have also contributed to such deviations even though there was no significant LD between all pairs of the 20 loci across the five chicken populations.

### Genetic Diversity Within the Populations

The total number of alleles per locus per population, the number of private alleles, and MNA per locus across the populations all indicated a high level of genetic diversity within the indigenous chicken populations of Sri Lanka. Furthermore, the highest MNA was exhibited by VC, NN, and LL, followed by CL, CR, FF, CC, and GR. Comparatively, low MNAs were recorded from other local chicken populations in the world, for example, Vietnamese local chickens (5.1) (Pham et al., 2013), Hungarian indigenous chicken breeds (2.9–4.2) (Bodzsar et al., 2009), French local breeds (6.6) (Berthouly et al., 2008), and Egyptian strains (4.92) (Eltanany et al., 2011). The higher number of alleles in VC, NN, and LL was due to the presence of private alleles that occurred at low frequencies within the populations and also due to the high number of observed alleles, owing to the free range management system, which allowed mixing of chickens among neighboring households (Bett et al., 2014).

All the chicken phenotypes from Sri Lanka had a high He, ranging from 0.61 to 0.72, which were similar to those of African and Asian scavenging chicken populations (Table 4). For example, the He of African scavenging chicken ranged from 0.53 to 0.66 (Leroy et al., 2012), of Vietnamese local chickens from 0.50 to 0.63 (Pham et al., 2013), and of traditional French breeds from 0.43 to 0.62 (Berthouly et al., 2008). In a study by Leroy et al. (2012), low He were reported for commercial lines compared with scavenging chicken populations, ranging from 0.29 to 0.48. However, a high He (0.639 ± 0.042) but a negative *F*_IS_ value (−0.016) were observed in CL (Table 4). Except in CL, positive *F*_IS_ values were present across all the village chicken populations, indicating non-random mating or the existence of population substructures with evidence of inbreeding within the populations. Comparatively, the high He and outbreeding of CL population could be due to the introduction of commercial strains to village farmers through interventions by the government and non-government organizations, aiming to uplift the chicken production for rural livelihoods.

Positive *F*_IS_ and high He estimates present in all village chicken populations can be due to the heterogeneity of the samples since they consisted of a mixture of phenotypes, which could be considered as genetic subdivision within the villages (e.g., the Wahlund effect) and non-random mating (Hedrick, 2013). Such subdivision may be explained by the observation of diverse phenotypes within the villages but specific chicken phenotypes often owned by different households. The 192 samples genotyped were selected from 818 samples representing all the phenotypes from the selected households where around 58% of households in these villages reared chickens with two or more phenotypes. Moreover, irrespective of the way of defining populations, i.e., based on geographical boundaries or based on phenotypic boundaries, a pattern of substructuring was also observed among the households, which is described in detail under the population structure section.

In addition, the high heterozygosity observed in this study indicated the individual variation within populations as a measure of allelic diversity. According to Nei (1987), heterozygosity is hardly affected by infrequent alleles at multi-allele loci. Therefore, the high heterozygosity observed in this study cannot be readily explained by the infrequent private alleles, which ranged from 2 to 9 in five villages and 1–2 in eight phenotypes. Every household had more than one phenotypic representation of birds included in our sampling. Thus, each population was a mixture of phenotypes with considerable allelic frequency. Nevertheless, the management system of free ranging supported a maximum and long run interaction among chickens from the neighboring households (Silva et al., 2014).

However, the expected and observed heterozygosity values were not significantly different (*p* > 0.05) in the five populations or in the eight phenotypes, suggesting a non-selective mating regime practiced among the Sri Lankan local chickens.

### Genetic Distance Between the Populations

The estimates of genetic distances clearly matched with the geographical distances among different village chicken populations. For example, village populations of TH and TB, which were located only around 43 km far apart showed a low genetic distance supported by a high bootstrap value (88%) compared with village populations of TH and OT, which were located around 120 km far apart and separated by the highest genetic distance (Figure 3). However, DM and TB village populations were from the two sites; their genetic distance were lower than the genetic distances between the villages of the same site (i.e., between DM and LA or OT; Table 5). Based on the estimates of phenotype-based populations, the NeighborNet tree (Figure 4) showed that the birds influenced by exotic chickens clustered together (CL, CR, and GR), while the crown chicken was separated further from all other phenotypic groups. Given the fact that crown (crest) is controlled by a single gene with incomplete dominant mode of inheritance, thus, the preference and selection of this phenotype could have similar influence on its separation as in the case of NN phenotypic group, where naked neck is also controlled by the same mode of inheritance. However, as depicted in the present study, the separation of CC from the remaining phenotypic groups could have been contributed by the crown characteristic as well as the association of crown gene with several other characteristics (Wang et al., 2012), for example, some modifier genes associated with different genetic architecture compared with the other phenotypic groups. However, it is interesting to note that the crown chicken found in the backyard system in Sri Lanka is different from the well-known Polish chicken, which is characterized by a “v”-shaped comb.

### Population Structure and Level of Admixture Among the Populations

**FIGURE 10:**
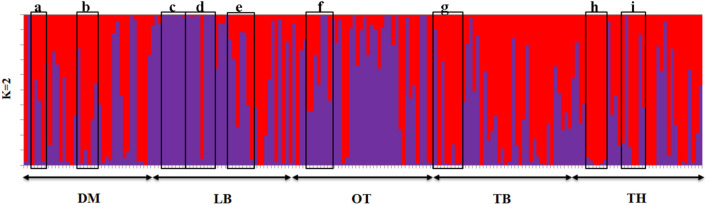
Households having more than five birds in the sampled population (from a–i). Phenotypes in (c) nacked neck and village chicken; (d) village chicken, frizzle feathered, commercial layer; (h) village chicken, naked neck, long legged, cross breds.

The graphical illustration based on structure analysis of chicken phenotypes did not show a distinguished population genetic structure, and the birds shared a highly admixed genetic background (Figure 5). This was also noticeable with the phenotypic diversity of these local chickens, where no distinguished color or comb pattern was observed (Liyanage et al., 2015). Moreover, a study by Silva et al. (2009) using mitochondrial DNA also revealed a similar finding within local chickens of Sri Lanka. However, the phenotypes of GR, CL, and CR tended to share a similar pattern of membership coefficient at *K* = 3 and 4, as represented by yellow color, where most of them had an exotic genetic background. Interestingly, several birds in naked neck phenotype showed the same clustering pattern along different *K* values, showing some substructure as illustrated in Figure 5. It was noticed that these NN birds were from the same farmer. To see a possible population substructuring pattern within the households, households with more than five birds in the sample were identified, and as given in Figure 10, several households had clear substructure within their chicken flocks though they had heterogeneous phenotypes. Therefore, a weak subclustering pattern was evident due to the mating system practiced following the restriction of limited or no exchange of breeding birds among the farmers.

Similarly, a high level of admixture was observed among the five geographical populations too (Figure 6), though LA showed a higher level of admixture of commercial strains. DM, TB, and TH villages also indicated a similar level of admixture among populations. The population genetic structure analysis suggested that the geographical isolation or closeness has influenced such similarities or differences in genetic structure within the village populations, for example, LA and OT villages in one site located close to each other compared with DM village. At the best *K* (*K* = 2), a similar population structure was observed in the two villages of LA and OT but leaving DM separated.

Similar results were also found in AMOVA where most variability was found at intra-individual level (89%) both within the populations defined by geographical boundaries and phenotypic boundaries. In addition, the observed genetic distances between the populations also confirmed the argument.

### Implications for Conservation and Upgrading in the Future

Local chickens of Sri Lanka do not have a distinguished population genetic structure, for example, a specific breed. However, a weak clustering was evident with the households due to the mating system practiced. One of the earlier studies by Gunaratne et al. (1993) has reported that, though backyard chickens were reared under the free range management system, there were limited or no exchange of breeding birds among farms. This could maximize the inbreeding within flocks of individual households, thus, leading to the formation of some substructure among the flocks.

Nevertheless, the local chickens possess a high genetic diversity in terms of total number of alleles and number of private alleles due to free range management coupled with mixed rearing of different phenotypes. According to our recent studies based on mitochondrial DNA and whole genome re-sequencing data generated from a worldwide sampling of domestic chickens, in all five wild Red Jungle Fowl (*Gallus gallus*) subspecies and other three wild Jungle Fowl species, it is evident that the backyard chicken populations of Sri Lanka rooted back to the Red Jungle Fowl that is not inhabitant in Sri Lanka, but not to the endemic species of Ceylon Jungle Fowl (*Gallus lafayetti*) of the country (Silva et al., 2009; Wang et al., 2020); therefore, the genetic diversity observed in Sri Lanka local chicken populations was invariably the result of contributions from different introductions in the past. It is known that Sri Lanka has been exposed to a variety of domestic animal species transported through trading in the past, owing to its critical location in the middle of the Indian Ocean connecting the sea routes between the East and the West. Thus, domestic chickens would have been a fair commodity for trade as well as a good protein source during long sea journeys since centuries back. Therefore, the evolutionary process of backyard chicken populations of the country has a long historical mixing of different chicken populations originating from several continents that may have contributed to both the high genetic and phenotypic diversity observed in this study.

The naked neck phenotype in study populations was superior in body weight, body circumference, keel length, and drum length (Bett et al., 2014). Furthermore, both naked neck and frizzle feathered genes were known to account for heat tolerance (Yunis and Cahaner, 1999). Nevertheless, farmers preferred the NN phenotype due to their higher carcass weight and higher egg production. As reported by Abeykoon et al. (2014), farmers in the study sites expressed their willingness to pay more for frizzle feathered phenotype, followed by crested chicken, thus, indicating their preference for local chickens over commercial strains. However, it is strange to note that, though there was a preference for certain phenotypes, majority of farmers do not practice a selective breeding program. Hence, the populations remained as an admixture group in village production system.

In contrast to the fast genetic progress that could be achieved by upgrading or crossbreeding programs, the diversity in the backyard chickens yields a steady and heterogeneous genetic base adapted to the low input/output smallholder system in Sri Lanka as in many parts of the developing world (FAO, 2010). While agreeing to the fact that the ancestral diversity that existed in the contributing populations leading to the admixture may be lost in the path of evolution of the backyard chicken populations, there is a curbing effect due to the absence of selection pressure or any directional selection, owing to the sociocultural reasons of farmers interwoven in the production system. Thus, the absence of common and directional selection could have been instrumental in preserving the rich diversity of contributing populations to a certain level, which otherwise could have been lost, resulting in high phenotypic and genetic diversity in the different populations studied.

The local chickens in Sri Lanka have been bred in the backyard low-input production systems for generations. Following this process, unique genetic variants could have evolved as adaptations to the climate and management conditions in Sri Lanka. Therefore, these birds can be used as a gene pool to maintain their major, specific genetic variants present among the backyard non-descript chicken populations in Sri Lanka. Since the genetic diversity existing in the populations studied are mainly constituted by individual-level variations, there is no strong population genetic structure formed within any of these populations. Therefore, they all show weak genetic fragmentations, even at household level due to the common management and breeding strategies practiced by farmers for generations. Accordingly, the present populations of local chickens in Sri Lanka serve the purpose of conservation through sustainable utilization, and more importantly, they could be considered as an ideal foundation for genetic improvement to establish breeds/lines to be adapted to particular environments and production systems in Sri Lanka.

The findings of this study confirm the genetic wealth conserved within the local chicken populations. The absence of population genetic structure is a result of the management and breeding regime commonly practiced in different localities of Sri Lanka. Therefore, we recommend that future strategies focus on sustainable development of this valuable resource with interventions appropriate to empower the existing operations of these village flocks in order to ensure that genetic diversity is maintained with time.

## Data Availability Statement

The original contributions presented in the study are included in the article/Supplementary Material, further inquiries can be directed to the corresponding author/s.

## Ethics Statement

The animal study was reviewed and approved by the Institutional Research and Ethics Committee (IREC) and Institutional Animal Care and Use Committees (IACUC) of the International Livestock Research Institute (ILRI), Nairobi, Kenya.

## Author Contributions

MI, JH, and PS conceptualized and designed the study. RL acquired the data. AS and JH analyzed the data. AS, JH, and PS interpreted the results. AS and PS drafted the article. AO, JH, and PS critically revised the article. MI, AO, JH, and PS gave the final approval of the version to be published. All authors contributed to the article and approved the submitted version.

## Conflict of Interest

The authors declare that the research was conducted in the absence of any commercial or financial relationships that could be construed as a potential conflict of interest.

## Publisher’s Note

All claims expressed in this article are solely those of the authors and do not necessarily represent those of their affiliated organizations, or those of the publisher, the editors and the reviewers. Any product that may be evaluated in this article, or claim that may be made by its manufacturer, is not guaranteed or endorsed by the publisher.

## Footnotes

1
https://spss.software.informer.com/13.0/

## Supplementary Material

Supplementary Table 1 Allele sizes of 20 microsatellite markers for eight phenotype-based populations: naked neck (NN), long legged (LL), crested or crown (CC), frizzle feathered (FF), Giriraj (GR), commercial layer (CL), crossbreds (CR), and non-descript village chicken (VC).

Supplementary Table 2 Allele sizes of 20 microsatellite markers for five village populations: Dematagama (DM), Labunoruwa (LA), Ooththupitiya (OT), Tabbowa (TB) and Thewanuwara (TH).
